# Epicatechin Protects Against Post-Cardiac Arrest Brain Injury in Aged Rats via NRG1-Mediated Suppression of Neuroinflammation

**DOI:** 10.3390/cimb47100793

**Published:** 2025-09-24

**Authors:** Hui-Hui Wang, Fan Huang, Zi-Long Du, Lu Xie

**Affiliations:** Department of Physiology Guangxi Medical University, Nanning 530021, China; 202210016@sr.gxmu.edu.cn (H.-H.W.); huangfan0816@sr.gxmu.edu.cn (F.H.); 202320008@sr.gxmu.edu.cn (Z.-L.D.)

**Keywords:** aging, Epicatechin, inflammation, cardiopulmonary resuscitation, Neuregulin-1, NF-κB

## Abstract

Chronic inflammation conducts an irreplaceable role in the aging process. More importantly, the impact is particularly significant in scenarios involving cardiac arrest and cardiopulmonary resuscitation (CA/CPR), where elderly individuals are inclined to suffer from more severe inflammatory injuries when compared to younger counterparts. Network pharmacology demonstrated a tight correlation between epicatechin (EC), aging, and the NRG1-NF-κB signaling pathway. With an aim to investigate whether EC suppressing inflammatory aging and alleviating post-CA/CPR brain injury is associated with the inhibition of the NRG1-NF-κB pathway, we established a model of naturally aged 21-month-old rats subjected to CA/CPR. A network pharmacology method was employed to pinpoint possible pathways that connect EC to neuroinflammation associated with aging. Sixty rats were randomly divided into three groups for feeding: a control group (pure water) and EC groups (EC was administered by gavage at doses of 1 mg/kg and 2 mg/kg respectively from the 12th month). Those groups underwent a CA/CPR procedure. At 24-h post-resuscitation, neurological scores, cortical pathology staining and assessments of neural injury were conducted. Expression levels of NRG1-NF-κB pathway-relevant inflammatory factors and proteins underwent systematic investigation by carrying out ELISA, RT-PCR, and Western blotting. In comparison with the 21-month-old groups treated with water, the 21-month-old groups treated with EC at 1 mg/kg and 2 mg/kg demonstrated decreased *β*-galactosidase staining, aging-correlated proteins and pro-inflammatory factors and NF-κB pathway-relevant proteins, as well as reinforced NRG1-ErbB4 expression. EC lessened inflammatory aging and mitigates post-CA/CPR brain injury in aged rats, associated with the inhibition of the NRG1-NF-κB pathway.

## 1. Introduction

The complex process of aging is characterized by persistent cellular and molecular degradation resulting from stress reactions, inflammation, metabolic disorders, and endocrine alterations [1,2]. This cumulative damage leads to irreversible physiological and pathological alterations, comprising structural and functional declines in organ systems. In 2000, Claudio Franceschi introduced the concept of ‘inflammatory aging’, where low-grade inflammation progressively exerts significant influences on a wide spectrum of tissues as age continues to increase [3]. This research has suggested an all-round elevation in proinflammatory and prooxidant transcriptional profiles and a significant reduction in growth and antioxidant gene profiles in the brains of aged rodents in contrast to those of young individuals [4]. Under oxidative stress, inflammatory mediators released from damaged or dying cells can give rise to phenotypic and functional changes in immune cells, such as the transition between M1/M2 microglia and A1/A2 astrocytes [5]. In some sense, the senescence-associated secretory phenotype (SASP) can be reckoned as an overall consequence of the cytokines, growth factors, and matrix metalloproteinases secreted by these activated cells [6]. Neurodegenerative diseases and cerebral ischemia are characterized by inflammation damage pathology [7], with the synergistic impact of aging and tissue trauma intensifying brain damage and deteriorating clinical outcomes in older patients.

Systemic circulatory failure results from cardiac arrest (CA), which is characterized by a sudden suspension of blood ejection and a cessation of heart mechanical activity [8]. CA triggers exceptionally high rates of mortality and disability, imposing a financial and emotional toll on families and society at large [9]. Notwithstanding the fact that timely cardiopulmonary resuscitation (CPR) can restore spontaneous circulation (ROSC), no more than approximately 10% of adult out-of-hospital cardiac arrest (OHCA) patients survive to hospital discharge, with over 80% suffering from myocardial and cerebral injuries and up to 60% enduring moderate-to-severe cognitive impairment three months subsequent to resuscitation [10,11,12,13]. The strong oxidative and inflammatory stress that occurs during the entire cerebral ischemia–reperfusion injury (CIRI) process leads to neuronal necroptosis and pyroptosis, which are the main cause of brain injury after ROSC [14,15]. Under such circumstances, attributable to the double blow of inflammatory stress resulting from age-associated inflammatory dysregulation combined with ischemia–reperfusion, elderly individuals exhibit greater vulnerability to more severe brain damage than their younger counterparts. [16,17]. For this reason, investigating anti-aging agents capable of suppressing chronic inflammation may strengthen resilience against disease and lower pathological damage.

EC is a prevalent dietary flavonoid that can be found in cereals, tea, cocoa, fruits, and vegetables [18]. EC has been demonstrated in recent research to have strong anti-inflammatory and antioxidant properties [19,20,21], to elevate nervous functions, and alleviate the symptoms of cardiovascular and cerebrovascular disorders. Nonetheless, it remains considerably ambiguous with regard to its specific mechanism of action and long-term effects in natural aging. Previous research employing OGD/R aging SH-SY5Y cells and MCAO models in 12-month-old rats revealed that EC prevents cerebral ischemia–reperfusion injury by augmenting glutathione (GSH) and superoxide dismutase (SOD) activity while lowering lactate dehydrogenase (LDH) and malondialdehyde (MDA) levels [22]. This academic and clinical endeavor was predominantly intended to delve into whether EC can mitigate aging-correlated neuroinflammation and lessen neuronal injury subsequent to CA/CPR. To start with, network pharmacology analysis is employed to predict EC-related anti-aging genes, unveiling their potential engagement in pathways such as the NF-κB pathway. Afterwards, naturally aging rats were raised until 21 months old. EC was administered by gavage from the 12th month to the 21st month (1 or 2 mg/kg). Upon the completion of the above steps, CA/CPR modeling was performed on aging rats. Aging markers, inflammatory factors, neural function and morphology in brain were measured to display the effects of EC on alleviating aging brain inflammation and the NRG1-NF-κB pathway, ameliorating CA/CPR neural outcomes in aged rats.

## 2. Materials and Methods

### 2.1. Experimental Methods of Network Pharmacology

To pinpoint potential targets of EC, we employed a multidatabase approach by employing TCMSP (https://old.tcmsp-e.com/tcmsp.php) (Date 14 July 2024), SuperPred (https://prediction.charite.de) (Date 14 July 2024) and PharmMapper (http://www.lilabecust.cn/pharmmapper/) (Date 14 July 2024). Aging-correlated genes were obtained from DisGeNET (https://www.disgenet.org/) (Date 14 July 2024) and GeneCards (https://www.genecards.org/) (Date 14 July 2024), after which duplicates were merged and removed. Gene nomenclature was standardized by employing UniProt ID mapping (https://www.uniprot.org) (Date 14 July 2024). Common targets shared between ECs and aging-correlated genes were identified by utilizing Venny 2.1 (https://bioinfogp.cnb.csic.es/tools/venny/) (Date 14 July 2024). STRING was utilized in the construction of a protein–protein interaction (PPI) network (https://cn.stringdb.org/) (Date 14 July 2024) for the species *Rattus norvegicus*, for which the confidence score was ≥ 0.4. Hub targets were defined as those with node degrees exceeding twice the median value. Kyoto Encyclopedia of Genes and Genomes (KEGG) pathway analysis and Gene Ontology (GO) functional enrichment analyses were conducted by employing DAVID (https://david.ncifcrf.gov/) (Date 14 July 2024) and the results were visualized with Weishengxin (http://www.bioinformatics.com.cn) (Date 14 July 2024). The 2D structure of EC (PubChem CID: 72276) was downloaded from PubChem (https://pubchem.ncbi.nlm.nih.gov/) (Date 14 July 2024). The crystal structure of NRG1 (PDB ID: pdb_00007mn5) was retrieved from the Protein Data Bank (https://www.rcsb.org/) (Date 14 July 2024). Prior to molecular docking, the NRG1 protein was processed in PyMOL 2.5 to remove ligands and water molecules. Hydrogen atoms were added, and the necessary parameters were set by utilizing AutoDock Tools 1.5.7. Molecular docking was subsequently performed by utilizing AutoDock, and binding affinities were evaluated on the basis of docking scores, with lower values illustrating more significant interactions. Final visualization of the docking results was conducted by adopting PyMOL.

### 2.2. Grouping and Administration of EC in Naturally Aged Rats

Sixty male specific-pathogen-free (SPF) Sprague-Dawley rats were acquired and acclimated in a controlled setting (12-h light/dark cycle, constant temperature, free access to food and water). At 12 months of age, the rats were randomized to receive oral gavages of either 1 mg/kg or 2 mg/kg epicatechin daily (*n* = 20 per group) or water (control group, *n* = 20). Every experimental approach was rigorously executed in strict adherence to national and international ethical standards for animal research and was authorized by Guangxi Medical University’s Animal Care and Use Committee (Approval No. 20230515; Date: 15 May 2023).

### 2.3. Grouping of CA/CPR in Aged Rats

The aforementioned three groups of naturally aged rats were categorized into Sham and CA/CPR randomly: 21-month-old water Sham group (21 Mo_Sham_Veh, *n* = 6), 21-month-old water CPR group, (21 Mo_CA/CPR_Veh, *n* = 6), 1 mg/kg EC sham group (21 Mo_Sham_EC1, *n* = 9), 1 mg/kg EC CPR group (21 Mo_CA/CPR_EC1, *n* = 6), 2 mg/kg EC Sham group (21 Mo_Sham_EC2, *n* = 10), 2 mg/kg EC CPR group (21 Mo_CA/CPR_EC2, *n* = 6). Apart from that, a separate cohort of 14 young Sprague-Dawley (SD) rats aged 3 months was included and assigned to either the Sham group (3 Mo_Sham_Veh, *n* = 6) or the CA/CPR group (3 Mo_CA/CPR_Veh, *n* = 8), with 6 animals surviving the procedure in the latter group. A table and a figure of experimental groups are shown in Supplementary Table S1 and Figure S1.

### 2.4. CA/CPR Model

The rats were given free access to water and fasted for 12 h prior to relevant operations. Subsequent to the determination of body weight, 2% sodium pentobarbital (Serve GmbH, Heidelberg, Germany) at a dose of 30 mg/kg was injected intraperitoneally to induce anesthesia. The process outlined by Chen et al. was used to create the CA model [23]. To put it simple, an F5 temporary pacemaker was orally inserted into the rat esophagus, using the incisors as a reference point, to a depth of approximately 7 cm near the sinoatrial node. The external end of the electrode was connected to an alternating current (AC) power source. Ventricular fibrillation (VF) was induced by delivering 12 V AC for 1 min until the mean arterial pressure (MAP) decreased to ≤20 mmHg. Subsequent to 5 min of untreated CA, CPR was initiated. A metronome was used to guide the manual chest compressions, which were carried out at a rate of 180 per minute and a compression depth of 25–30% of the anteroposterior thoracic diameter. It is particularly noteworthy that the same investigator should guarantee uniform chest compression-to-release ratios throughout the entire procedure. Following endotracheal intubation, mechanical ventilation (DH-150, Department of Medical Instruments, ZheJiang University, Hangzhou, China) was started with a tidal volume of 6 mL/kg, a respiratory rate of 70 breaths per min, and zero positive end-expiratory pressure (PEEP). One minute after the initiation of CPR, epinephrine(Grand Pharma Wuhan, China) (20 μg/kg) was administered by utilizing the left femoral venous catheter. The return of a supraventricular rhythm (e.g., sinus, atrial, or junctional rhythm) combined with a mean aortic pressure exceeding than 60 mmHg sustained for more than 1 min is defined as return of ROSC. A heating pad was employed to maintain the rat’s rectal temperature at 37 ± 0.5 °C throughout the surgery, from anesthesia to recovery. Following acclimatization, the animals were relocated to sanitary cages with dry bedding and kept at 27 °C in a tranquil environment.

### 2.5. Survival Observation and Neurological Evaluation

The neurological results and survival rates were evaluated 24 h upon the completion of resuscitation. In accordance with Jia et al., the neurological disability score (NDS) was employed to assess neurological deficiencies [24,25]. The NDS, with scores spanning from 0 (death) to 80 (no discernible neurological damage), assesses consciousness, motor function, sensory reactivity, respiration, and behavior. Two investigators independently conducted the assessments and achieved a unanimous agreement on every score.

### 2.6. β-Galactosidase Staining

A cryostat was utilized to segment fresh brain tissues for *β*-galactosidase staining (Beyotime C0602 Beijing Huaxia Ocean Technology Co., Ltd., Beijing, China) upon their rapid freezing in liquid nitrogen. In brief, the sections were fixed at room temperature for 15 min following three PBS washes. The slices were incubated with a freshly made staining solution that contained Staining Solutions A-C and X-Gal throughout the entire night at 37 °C after fixation. To prevent evaporation, the slides were sealed throughout the process of incubation. The stained sections underwent imaging under a light microscope and subsequent analysis by employing ImageJ software (version: 1.53).

### 2.7. Hematoxylin and Eosin Staining

Three anesthetized rats with 24 h neurological deficit scores were randomly selected from the Sham and ROSC groups. Following transcardial perfusion with 4% paraformaldehyde, coronal brain sections (4 mm thick) were obtained 1 mm posterior to the optic chiasm and postfixed in the same fixative for paraffin embedding. After being cut with a Leica RW2265 microtome (Leica Company in Germany, Wetzlar, Germany) and stained with hematoxylin and eosin (H&E G1076 Wuhan servicebio technology Co., Ltd., Wuhan, China), 3–5 μm thick tissue slices were observed at 200× magnification by utilizing an Olympus BX53 optical microscope (Olympus, Tokyo, Japan). ImageJ software (v1.33, NIH, Bethesda, MD, USA) was used to evaluate five randomly selected fields per section.

### 2.8. Transmission Electron Microscopy (TEM)

The rats were fixed in 2.5% glutaraldehyde (Wuhan servicebio technology Co., Ltd., Wuhan, China) subsequent to transcardial perfusion with saline (Biosharp, Hefei City, China) or 4% paraformaldehyde (Biosharp, BL539A, Hefei City, China). Rapid brain dissection was followed by the postfixation of 1 mm^3^ of tissue blocks from the right cerebral cortex in 2.5% glutaraldehyde. A Leica EM UC7 ultramicrotome (Leica Company in Germany, Wetzlar, Germany) was employed to cut ultrathin sections (80 nm) following dehydration and embedding. Sections were observed by employing an electron microscope (H-7650, Hitachi, Tokyo, Japan) after being stained with uranyl acetate and citrate. A scoring system was employed for blinded assessment of mitochondrial damage [26,27]. An evaluation was conducted on five randomly chosen grids. Scores ranged from grade 0 (well-organized cristae) to grade 4 (severe swelling with disrupted cristae and ruptured membranes).

### 2.9. Serum Levels of TNF-α, IL-1β, IL-6 and IL-10 Were Quantified by Adopting Enzyme-Linked Immunosorbent Assay (ELISA)

An ELISA was adopted to measure the levels of TNF-α, IL-1β, IL-6 and IL-10 in the serum. Pentobarbital sodium (Serve GmbH, Heidelberg, Germany) (60 mg/kg) was administered intraperitoneally to five rats in each group to induce anesthesia prior to the blood collection. Following a neurological impairment assessment and 24 h subsequent to resuscitation, blood samples were collected from the left ventricle. Centrifugation was adopted to separate the serum, which was subsequently stored at −80 °C until analysis. Commercial ELISA kits (Wuhan Fine Biotech Co., Ltd., Wuhan, China) were used in accordance with the manufacturer’s instructions. Cytokine concentrations were computed by employing standard curves generated by Curve Expert 1.3 software (version: Curve Expert 1.3) adjusted for sample dilution, and the absorbance at 450 nm was quantified by employing a microplate reader.

### 2.10. RNA Extraction and RT-qPCR

Cortical tissue was treated with TRIzol^®^ (Invitrogen, Lot No: 15596026CN, Thermo Fisher, CA, USA) to extract total RNA. One microgram of RNA was converted into cDNA by employing an RT-qPCR kit (Code NO: RR092B, Takara, Dalian Bao Biological Co., Ltd., Dalian, China). Under standard cycling conditions (95 °C for 5 s, 60 °C for 10 s, 40 cycles), qPCR was subsequently performed on a Step One Plus system (Applied Biosystems, Serial number: 2720501097, Thermo Fisher Scientific, Singapore). The 2^−ΔΔCT^ technique was employed to comprehensively assess expression levels after normalization to β-actin. The qPCR primers used for p21, p53, NRG1, ErbB4, IκBα and p65 are shown in Supplementary Table S2.

### 2.11. Western Blot Analysis

Proteins were separated via 10% or 15% SDS-PAGE upon homogenization (Code No: LK407-20, Epizyme Biotech, Shanghai, China) of the cortex tissue (20 mg) in 200 μL of lysis buffer (RIPA:PMSF:protein phosphatase inhibitor, 100:1:1). Subsequent to the above steps, the proteins were placed onto a PVDF membrane (Merck Millipore Limited, Tullagreen, Castletownroche, County Cork, Ireland; pore size: 0.22 μm). The membrane was blocked for 1 h with 5% skim milk and then incubated overnight at 4 °C with primary antibodies against ErbB4 (1:500, 19943–1-AP, Proteintech, Rosemont, IL, USA), NRG1 (1:500, 10527–1-AP, Proteintech), p21 (1:200, Sc-6246, Santa Cruz, Dallas, TX, USA), p53 (1:200, Sc-393031, Santa Cruz), p-IκBα (1:1000, 2859T, CST, Danvers, MA, USA), IκBα (1:1000, 4814T, CST), p-NF-κB-p65 (1:1000, 3033T, CST), NF-κB-p65 (1:1000, 8242T, CST), BCL2 (1:2000, AB196495, Abcam, Cambridge, UK), β-Tubulin (1:1000, AB108342, Abcam), and GAPDH (1:1000, 2118S, CST). Following a washing process, the membrane was incubated with fluorescent secondary antibodies (rabbit, 1:5000; LOT#D30627-01; LI-COR; mouse, 1:5000; XE325771) at room temperature for 1 h. Protein bands were ultimately detected with a LI-COR Odyssey Scanner (Gene Company limited, Shanghai, China), and ImageJ software (v1.33, NIH, Bethesda, MD, USA) was employed to quantify the bands.

### 2.12. Statistical Analyses

The associated pictures and figure legends specify the number of rats used in each experiment. SPSS 24.0 software (SPSS Inc., Chicago, IL, USA) was utilized to analyze the data, and the results are presented as the means ± standard deviations (SDs). The following parameters were compared across several groups by adopting one-way analysis of variance (ANOVA): CPR duration, neurological deficit score (NDS), SA-*β*-gal staining, and all Western blot, qPCR and inflammatory factor expression data. The Kruskal–Wallis test was adopted to compare the survival rates of various groups. The definition of statistical significance was *p* < 0.05. The experimenters were blinded to group assignments throughout the process of data analysis to minimize potential experimenter bias. Graph layouts were achieved with GraphPad Prism9 (version: 9.4.0, GraphPad Software, Inc., San Diego, CA, USA).

## 3. Results

### 3.1. Network Pharmacology Provides a Theoretical Basis for Understanding the Interaction Between ECs and Aging-Related Signaling Pathways

**Figure 1:**
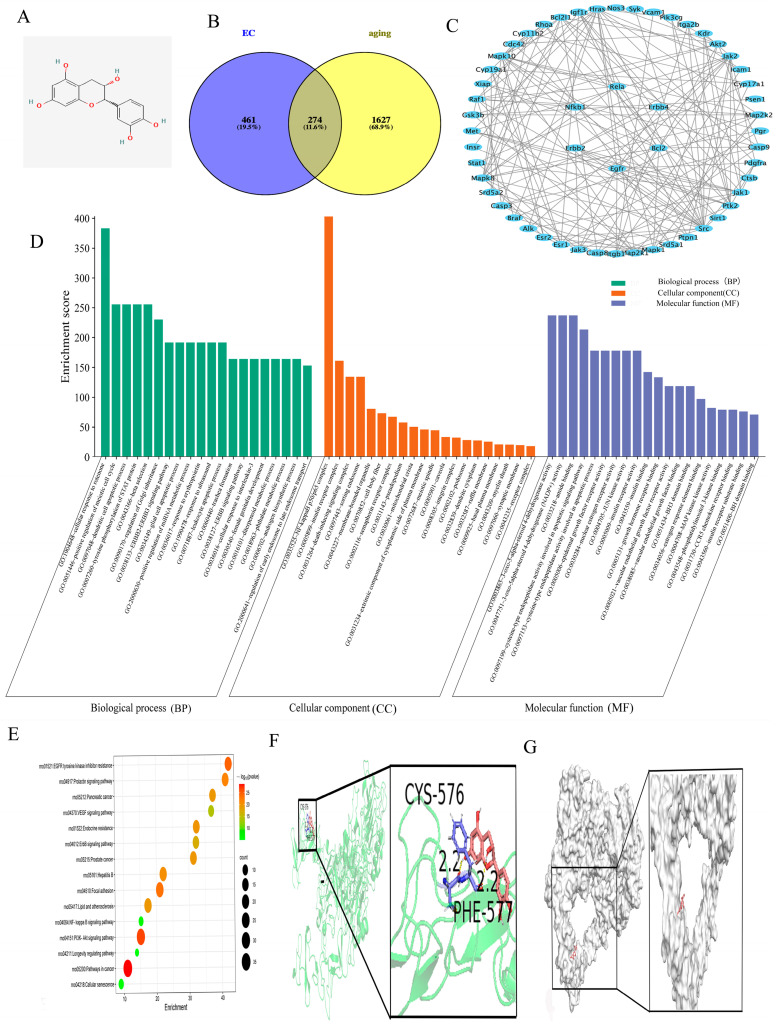
Results of network pharmacology and molecular docking. (**A**) 2D structural diagram of EC. The red O represents the oxygen element in the 2D structure of epicatechin. (**B**) Venn diagram illustrating the overlap between EC targets and aging—related genes. (**C**) Core components were identified by adopting Cytoscape 3.10.2 grounded in a median degree greater than 2, ultimately bringing about 54 paramount targets, comprising EGFR, ErbB4, NFKB1 and BCL2. (**D**) GO analysis of the 54 core targets revealed 15 enriched phrases related to molecular functions (MFs), cellular components (CCs) and biological processes (BPs). (**E**) KEGG pathway enrichment analysis unveiled critical pathways tightly correlated with anti-aging effects, such as the EGF receptor tyrosine kinase inhibitor resistance pathway, the PI3K-Akt signaling pathway and the cancer proteoglycan pathway. (**F**) As molecular docking results suggested, EC binds effectively to Neuregulin-1 (NRG1), illustrating a binding affinity of −2.68 kcal/mol. (**G**) Visualization via PyMOL confirmed stable interactions between EC and NRG1, which adequately demonstrates favorable binding activity.

The 2D structure of EC is revealed in Figure 1A. As described in Section 2.1, potential active targets of EC and aging-related genes were identified. A Venn diagram revealed 54 overlapping targets, comprising paramount molecules such as EGFR, ErbB4, NFKB1 and BCL2 (Figure 1B,C). Gene Ontology (GO) analysis revealed that these targets were involved mainly in cellular response mechanisms, apoptosis signaling and NF-κB complex regulation (Figure 1D). KEGG pathway enrichment revealed pronounced enrichment of the PI3K-Akt signaling pathway, the EGF receptor tyrosine kinase inhibitor resistance pathway and the cellular senescence pathway (Figure 1E). Molecular docking demonstrated a significant binding affinity between EC and neuregulin 1 (NRG1), with a binding energy of −2.68 kcal/mol (Figure 1F). PyMOL visualization validated stable interactions between EC and NRG1, hinting at a feasible molecular mechanism underlying their functional relationship (Figure 1G).

### 3.2. EC Increased Neurological Deficit Scores and Survival Rates After CA/CPR

**Figure 2:**
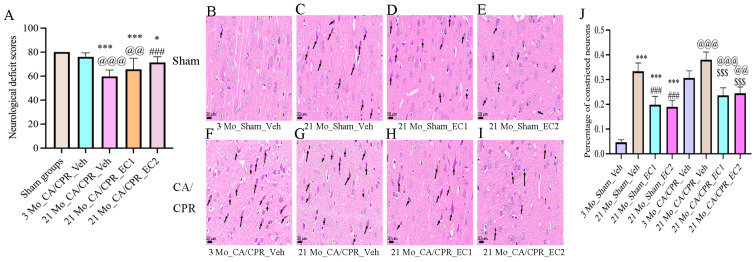
Neurological function score and HE staining of the cerebral cortex in rats. (**A**) The neurological deficit scores are presented as the Mean ± SD. * *p* < 0.05, *** *p* < 0.001 vs. Sham groups; ^###^ *p* < 0.001 vs. 21 Mo_CA/CPR_Veh group; ^@@^ *p* < 0.01, ^@@@^ *p* < 0.001 vs. 3 Mo_CA/CPR_Veh group. (**B**–**I**) HE staining of the cerebral cortex after ROSC for 24 h. Scale bar:20 μm (200×); The black arrows indicate deeply stained constricted neurons. (**J**) Statistical chart showing the percentage of pyknotic neurons. All data are presented as the Mean ± SD. *** *p* < 0.001 vs. 3 Mo_Sham_Veh group; ^###^ *p* < 0.001 vs. 21 Mo_Sham_Veh group; ^@@^ *p* < 0.01, ^@@@^ *p* < 0.001 vs. 3 Mo_CA/CPR_Veh group; ^$$$^ *p* < 0.001 vs. 21 Mo_CA/CPR_Veh group.

As illustrated in Table 1, the 21 Mo_CA/CPR_Veh group presented lower survival rates than the sham group. There were no discernible changes between the 3 Mo_CA/CPR_Veh, 21 Mo_CA/CPR_EC1 and 21 Mo_CA/CPR_EC2 groups. As demonstrated in Figure 2A, the neurological function score of the 21 Mo_CA/CPR_Veh group was considerably lower than that of the 21 Mo_CA/CPR_EC2 and 3 Mo_CA/CPR_Veh groups. Scores were higher for the Sham group than for the 21 Mo_CA/CPR_Veh and 21 Mo_CA/CPR_EC1 groups. Notwithstanding the fact that continuous EC delivery betters neurological outcomes in elderly animals subsequent to cerebral ischemia–reperfusion injury, our data suggest that, in contrast to young rats, naturally aged rats exhibit heightened susceptibility to such injury. Histological analysis via HE staining is summarized in Figure 2B–J: Figure 2B (3 Mo_Sham_Veh group): Normal cortical architecture with well-organized neurons and uniform staining. Figure 2C (21 Mo_Sham_Veh group): 33.26 ± 3.3% of the neurons presented pyknosis, with irregular morphology and elevated nuclear staining intensity. Figure 2D (21 Mo_Sham_EC1 group): Disordered neuronal arrangement and uneven staining; 21.14 ± 3.7% pyknotic neurons with lowered cell volume. Figure 2E (21 Mo_Sham_EC2 group): Mild cortical damage and relatively preserved neuronal organization; 18.43 ± 2.6% pyknotic neurons. Figure 2F (3 Mo_CA/CPR_Veh group): Significant augment in deeply stained neurons (31.31 ± 2.5%), which obviously suggests acute injury. Although this percentage remained greater than that in the 3 Mo_CA/CPR_Veh group, pyknotic neurons lowered to 38.52± 2.3%. Figure 2H (21 Mo_CA/CPR_EC1 group): Presence of deep neuronal staining and pyknosis; 24.07 ± 2.6% pyknotic neurons. Figure 2I (21 Mo_CA/CPR_EC2 group): Further reduction in pyknotic neurons (24.86 ± 2.7%). In contrast to the 3 Mo_Sham_Veh group, the 21 Mo_Sham_Veh, 21 Mo_Sham_EC1 and 21 Mo_Sham_EC2 groups presented more pyknotic neurons. In comparison with the 21 Mo_Sham_Veh group, the 21 Mo_Sham_EC1 and 21 Mo_Sham_EC2 groups presented fewer pyknotic neurons. There were more pyknotic neurons in the 21 Mo_CA/CPR_Veh group than in the 3 Mo_CA/CPR_Veh group. Neuronal pyknosis was considerably lower in the 21 Mo_CA/CPR_EC1 and 21Mo_CA/CPR_EC2 groups than in the 21 Mo_CA/CPR_Veh group.

### 3.3. EC Improved the Structure of Neurons After CA/CPR

**Figure 3:**
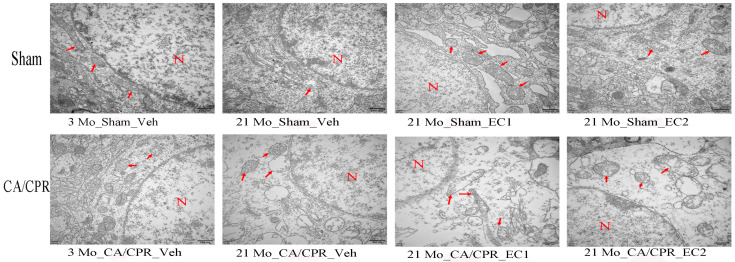
Electron microscopy of neuronal organelles. Sample transmission electron microscopy (TEM) images of cerebral cortex neurons at 24 h after ROSC. Scale bar 500 nm (30,000×). Red arrows indicate mitochondria; N indicates nucleus.

In line with ultra-structural studies, the aging groups presented significant mitochondrial damage. Figure 3: In comparison with those in the 3 Mo_Sham_Veh, 21 Mo_Sham_EC1 and 21 Mo_Sham_EC2, the mitochondria in the 21 Mo_Sham_Veh group presented vacuolar degeneration, lowered electron density and matrix thinning. In the CA/CPR group, the mitochondria in the 21 Mo_CA/CPR_Veh subgroup presented severe structural alterations, comprising crista disintegration, rupture and loss, as well as disrupted neuronal membranes. Comparison of mitochondrial structure between CA/CPR aging group (21 Mo_CA/CPR_Veh, 21 Mo_CA/CPR_EC1 and 21 Mo_CA/CPR_EC2) with that of sham aging group (21 Mo_Sham_Veh, 21 Mo_Sham_EC1 and 21 Mo_Sham_EC2) demonstrated that CA/CPR group shows more mitochondrial vacuolization, crista disorganization and membrane rupture, along with more pronounced neuronal membrane incompleteness, which evidently demonstrated exacerbated mitochondrial and cellular injury in aged animals subsequent to CA/CPR.

### 3.4. EC Down-Regulated Aging Indicators

**Figure 4:**
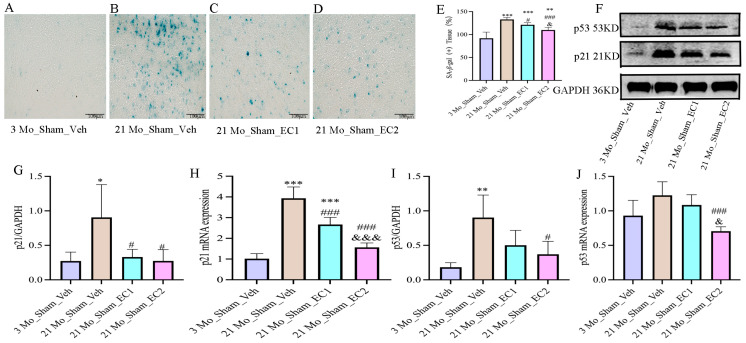
SA-*β*-gal staining and the protein and mRNA expression levels of p53 and p21. (**A**–**D**) A representation of the proportion of SA-*β*-gal-stained regions. Scale bar: 100 μm (400×). (**E**) SA-*β*-gal (+) Tissue (%). (**F**) The WB bands of the senescence-related proteins p21 and p53. (**G**–**J**) The statistical map of p21 and p53 WB and mRNA statistical analysis. All data are presented as the Mean ± SD. * *p* < 0.05, ** *p* < 0.01, *** *p* < 0.001 vs. 3 Mo_Sham_Veh group; ^#^ *p* < 0.05, ^###^ *p* < 0.001 vs. 21 Mo_Sham_Veh group; ^&^ *p* < 0.05, ^&&&^ *p* < 0.001 vs. 21 Mo_Sham_EC1 group.

Heightened cellular senescence is indicated by elevated staining intensity of *β*-galactosidase (SA-*β*-gal). Figure 4A–D: Notably, significantly greater staining was detected in the 21 Mo_Sham_Veh group compared to the 3 Mo_Sham_Veh and 21 Mo_Sham_EC2 groups. EC may lessen age-related senescence, as evidenced by the 3 Mo_Sham_Veh group’s lower staining than the 21 Mo_Sham_EC1 and 21 Mo_Sham_EC2 groups did. Figure 4F–J: In comparison with the 21 Mo_Sham_EC2 group, the 21 Mo_Sham_Veh group also presented more significant p53 and p21 protein levels and strengthened p21 mRNA expression. Apart from that, p21 mRNA was up-regulated in the 21 Mo_Sham_EC1 group in contrast to the 21 Mo_Sham_EC2 group. Altogether, the above findings suggest that activation of the p53/p21 senescence pathway throughout the process of aging can be partially attenuated by EC treatment. It is tremendously noteworthy that the 2 mg/kg dose of EC appeared to be more effective than the 1 mg/kg dose.

### 3.5. EC Attenuated Inflammatory Aging

**Figure 5:**
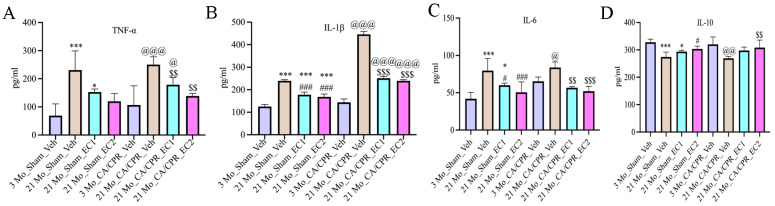
ELISA results for TNF-1β, IL-6, and IL-10, among other inflammatory factors. (**A**–**D**) All data are presented as the Mean ± SD. * *p* < 0.05, *** *p* < 0.001 vs. 3 Mo_Sham_Veh; ^#^ *p* < 0.05, ^###^ *p* < 0.001 vs. 21 Mo_Sham_Veh group; ^@^ *p* < 0.05, ^@@^ *p* < 0.01, ^@@@^ *p* < 0.001 vs. 3 Mo_CA/CPR_Veh group; ^$$^ *p* < 0.01, ^$$$^ *p* < 0.001 vs. 21 Mo_CA/CPR_Veh group.

The anti-inflammatory cytokine IL-10 was less pronounced, and the proinflammatory cytokines TNF-α, IL-1β and IL-6 were greater in the 21 Mo_Sham_Veh group than in the 3 Mo_Sham_Veh group. On the other hand, the 21 Mo_Sham_EC2 group displayed more significant levels of IL-10 and lower levels of IL-1β and IL-6 than the 21 Mo_Sham_Veh group. Figure 5A–D: In contrast to the 3 Mo_CA/CPR_Veh group, the 21 Mo_CA/CPR_Veh group in the CA/CPR group presented considerably higher levels of TNF-α and IL-1β, whereas the level of IL-10 was less significant. It is tremendously noteworthy that in comparison with the 21 Mo_CA/CPR_EC2 group, the 21 Mo_CA/CPR_EC2 group presented lowered IL-1β and IL-6 levels, with a concomitant heightening IL-10.

### 3.6. EC Modulates the NRG1-NF-κB Pathway in the Context of Aging-Related Inflammation

**Figure 6:**
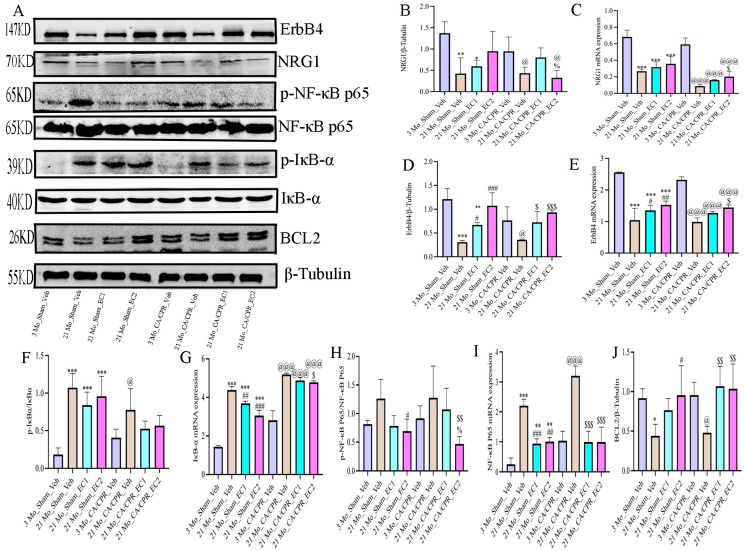
The results of Western blot (WB) and mRNA expression analyses of NRG1, ErbB4, p-IKKα, p-p65 and BCL2 in both the sham and CA/CPR groups.(**A**) NRG1-NF-κB signaling pathway-related proteins; (**B**) NRG1 WB data analysis; (**C**) expression profile of NRG1 mRNA; (**D**) ErbB4 WB data analysis; (**E**) ErbB4 mRNA expression levels; (**F**) p-IκBα/IκBα WB data analysis; (**G**) expression profile of IκBα mRNA; (**H**) p-NF-κBp65/NF-κBp65 WB data analysis; (**I**) NF-κBp65 mRNA expression levels; (**J**) BCL2 WB data analysis. All data are presented as the Mean ± SD. * *p* < 0.05, ** *p* < 0.01, *** *p* < 0.001 vs. 3 Mo_Sham_Veh; ^#^ *p* < 0.05, ^##^ *p* < 0.01, ^###^ *p* < 0.001 vs. 21 Mo_Sham_Veh group; ^@^ *p* < 0.05, ^@@@^ *p* < 0.001 vs. 3 Mo_CA/CPR_Veh group; ^$^ *p* < 0.05, ^$$^ *p* < 0.01, ^$$$^ *p* < 0.001 vs. 21 Mo_CA/CPR_Veh group; ^%^ *p* < 0.05 vs. 21 Mo_CA/CPR_EC1 group.

Figure 6 A depicts representative Western blot images of NRG1, ErbB4, p-IκBα, IκBα, p-NF-κBp65, NF-κBp65 and BCL2. Age-related downregulation of this signaling axis was demonstrated by the considerably lower expression of both NRG1 and ErbB4 in the 21 Mo_Sham_Veh group than in the 3 Mo_Sham_Veh group in the Sham group (Figure 6B,D,F,H). Concurrently, there was a significant elevation in the ratios of p-IκBα/IκBα and p-NF-κBp65/NF-κBp65, which apparently illustrated that the NF-κB pathway was activated throughout the process of normal aging. Both the 21 Mo_Sham_EC1 and 21 Mo_Sham_EC2 groups presented higher levels of NRG1 and ErbB4 than the 21 Mo_Sham_Veh group did, suggesting that long-term EC administration may be advantageous for the repeated expression of the aforementioned proteins among elderly rats. Apart from that, the phosphorylation levels of IκBα and NF-κBp65 were lessened in these groups, illustrating that EC conducts a pivotal role in suppressing NF-κB signaling and alleviating age-associated inflammation. A similar pattern was observed in the CA/CPR groups (Figure 6B,D,F,H). NRG1 and ErbB4 expression was lower in the 21 Mo_CA/CPR_Veh group than in the 3 Mo_CA/CPR_Veh group, while the p-IκBα/IκBα and p-NF-κBp65/NF-κBp65 ratios were greater. NRG1 and ErbB4 expression, nevertheless, was more significant in the EC-treated groups (21 Mo_CA/CPR_EC1 and 21 Mo_CA/CPR_EC2) than in the 21 Mo_CA/CPR_Veh group. Furthermore, the 21 Mo_CA/CPR_EC2 group presented a pronounced abatement in both phosphorylation markers in comparison with the 21 Mo_CA/CPR_Veh group, illustrating that EC inhibits NF-κB activation subsequent to CA/CPR in elderly animals. As displayed in Figure 6C,E,G,I, the mRNA expression levels of NRG1, ErbB4, p-IκBα and p-p65 corresponded with the corresponding protein data for both the CA/CPR and Sham groups. As depicted in Figure 6J, BCL2 expression was up-regulated in the 21 Mo_CA/CPR_EC1 and 21 Mo_CA/CPR_EC2 groups in contrast to that in the 21 Mo_CA/CPR_Veh group, demonstrating that EC treatment reinforced antiapoptotic activity. On top of that, BCL2 expression was considerably lower in the 21 Mo_Sham_Veh group than in the 21 Mo_Sham_EC2 group, revealing that oral gavage of EC for an extended period provides more satisfactory protection against apoptosis than does ordinary water consumption.

## 4. Discussion

The GO term analysis revealed that these targets were involved in a wide range of BPs, such as cellular responses, CCs comprising the NF-κB complex and MFs encompassing epidermal growth factor receptor activity, revealing that EC interacts with multiple pathways and aging-correlated mechanisms. KEGG pathway enrichment analysis further identified several aging-correlated signaling pathways, comprising the EGFR tyrosine kinase inhibitor resistance pathway, cellular senescence pathway, NF-κB signaling pathway and longevity regulation pathway. PPI network analysis identified pivotal targets such as EGFR, ErbB4, NFKB1, RELA and BCL2 holding pivotal roles within the interaction network. As further validated by employing molecular docking technology, the binding energy between EC and Neuregulin-1 (NRG1) is −2.68 kcal/mol, illustrating a favorable affinity between EC and NRG1. This finding underscores the validity and dependability of our network pharmacology predictions, offering a robust foundation for subsequent investigations. A multitude of domestic and international studies have reported that NRG1, a neurotrophic growth factor, participates in the regulation of inflammation via the NF-κB signaling pathway [28]. It is pivotal to mention that its expression declines with advancing age and has been linked to the process of inflammation. Rooted in these groundbreaking findings, the existing research probed further into the effects of EC on the NRG1-NF-κB pathway to explore its potential mechanisms of action.

In this study, we systematically dug into the effects of EC on inflammatory aging. Considering that oral administration is the preferred method of long-term use for health care drugs, this experiment adopted gavage. As evidenced by our experimental findings, the expression of SA-*β*-gal, p53 and p21 in 21-month-old rats was more significant than those in 3-month-old rats. Among rats of the same age (21 months), these markers were lower in the EC-treated group in comparison with the water-treated control group, demonstrating that long-term oral administration of EC exerts anti-aging effects. Elevated SA-*β*-gal activity and strengthened expression of cell cycle inhibitors, comprising p21, p53 and p16, are hallmarks of cellular senescence. As already suggested by relevant studies, the up-regulation of p21 is a crucial regulatory node in aging, which can not only be attributable to its non-negligible role in blocking cyclin-dependent kinases, but also can be ascribable to the fact that it modulates the transcription of genes essential for the senescence phenotype [29]. Moreover, p53 activation has been implicated in tumor-induced senescence [30]. Our findings not only reinforce this hypothesis but also, for the first time, reveal that EC orchestrate p21 expression throughout the natural aging trajectory. Concurrently, the severity of immune dysfunction escalates progressively over the aging continuum. As we previously studied and illustrated, long-term administration of EC can delay inflammatory aging in the brains of aged rats (18 months old) by reinforcing the activation of the Wnt/β-catenin signaling pathway. The present study extended the anti-aging effect of EC in 21-month-old rats, which demonstrated a significant augment in the expression levels of pro-inflammatory cytokines IL-1β and IL-6 with a trend toward elevated TNF-α levels, whereas a decreasing trend of the anti-inflammatory cytokine IL-10 in comparison with 3-month-old rats. These inflammatory phenotypes in 21-month-old rats were attenuated by EC administration.

Inflammation was identified as a paramount contributor to pathological damage [31,32]. Chronic inflammation compromises host immune defenses and exacerbates the inflammatory storm subsequent to CA/CPR, contributing to cerebral injury. As evidenced by our analytical findings, CA/CPR tremendously reinforced the expression of pro-inflammatory cytokines TNF-α, IL-1β and IL-6 in both 3-month-old and 21-month-old rats in contrast to age-matched sham controls. It is pivotal to mention that among the 21-month-old animals, these inflammatory markers were markedly lower in the EC-treated group than in the water-treated control group. Furthermore, TNF-α and IL-1β levels in 21-month-old CA/CPR rats were higher than those observed in their 3-month-old counterparts. In contrast to the water-treated 21-month-old CA/CPR group, the EC-treated group exhibited lessened expression of TNF-α, IL-1β and IL-6. Collectively, these findings suggest that long-term oral administration of EC attenuates systemic inflammation in aged rats, augments resilience against cerebral inflammatory injury and thereby betters neurological outcomes and brain histopathology after CPR. We found that EC-treated rats exhibited immensely strengthened neurological function scores 24 h after resuscitation in contrast to the water-treated group. Histological analysis by conducting H&E staining revealed better-preserved neuronal morphology in the EC-treated group, while electron microscopy suggested reinforced ultra-structural integrity of neurons, thus bringing about strengthened neurological function. Because 24 h is the critical pathological window for cerebral ischemic injury, this experiment only selected this single time point. Future studies will include 72 h and 7 d endpoints to assess the effects of EC on secondary injury and long-term recovery.

Numerous inflammatory mediators are primarily regulated by the NF-κB signaling pathway. It is universally acknowledged that activation of the NF-κB (nuclear factor kappa B) pathway in response to ischemia–reperfusion injury is triggered by a wide spectrum of stimuli and conducts a central role in regulating inflammatory gene expression. As already revealed by domestic and international studies, ischemia–reperfusion activates NF-κB, which subsequently facilitates the release of pro-inflammatory cytokines such as TNF-α and IL-6, exacerbating the inflammatory cascade and ultimately triggering cellular damage [33]. A critical step in NF-κB pathway activation is the degradation of IκB proteins, which normally bind to NF-κB and inhibit its transcriptional activity. Upon stimulation, IκB undergoes ubiquitination and proteasomal degradation, thereby enabling NF-κB to translocate into the nucleus and initiate the transcription of target genes. A systematic investigation on cerebral ischemia–reperfusion injury has forcefully demonstrated that NF-κB nuclear translocation is tightly correlated with neuroinflammation. Inhibiting NF-κB activation has been proven to lower neuronal cell death and suppress inflammatory responses, thereby offering neuroprotective benefits [32,33]. Since these inflammatory mediators are under the regulation of the NF-κB signaling pathway, these findings suggest that EC exerts its anti-aging effects, at least partially, through anti-inflammatory mechanisms. As validated by massive in-vitro and in-vivo studies on a wide spectrum of tissue types, EC can lower inflammation by inhibiting the NF-κB signaling pathway [19,20,34]. Throughout the process of cellular senescence, persistent activation of NF-κB accelerates the generation of pro-inflammatory cytokines such as IL-1β, IL-6 and TNF-α, which in turn amplify the inflammatory response, forming a self-perpetuating cycle that accelerates aging [35,36]. Relevant studies have suggested that sustained NF-κB activity can expedite cellular senescence by reinforcing the expression of pro-inflammatory genes. It is paramount to note that certain flavonoids and polyphenolic compounds have been proven to directly modulate the NF-κB signaling pathway by inhibiting the activity of the p65 subunit, thereby conducting an inhibitory role in the expression and secretion of inflammatory cytokines. This mechanism highlights the potential of plant-derived extracts not only as sources for anti-inflammatory drugs but also as leads for developing novel therapeutic strategies targeting chronic inflammation and aging-correlated diseases [37,38]. In this study, we examined the expression of proteins bound up with the NRG1-NF-κB signaling pathway. As evidently demonstrated by our experimental findings, CA/CPR lowered the expression of NRG1 and ErbB4 while heightening the phosphorylation levels of IκBα and NF-κB p65 in both 3-month-old and 21-month-old rats in comparison with age-matched sham controls. It is tremendously noteworthy that among 21-month-old rats, the EC-treated group exhibited higher NRG1 and ErbB4 expression, lower p-IκBα/IκBα and p-NF-κBp65/NF-κBp65 ratios, and strengthened BCL2 expression in contrast to the water-treated group. Moreover, 21-month-old rats exhibited lower NRG1 and ErbB4 levels and higher activation of the NF-κB pathway than 3-month-old rats, demonstrating that aging is tightly correlated with down-regulation of the NRG1-ErbB4 axis and enhanced NF-κB signaling. Similarly, 21-month-old CA/CPR rats illustrated more dramatic suppression of NRG1 and ErbB4 and more significant NF-κB activation than their 3-month-old counterparts. In contrast to the water-treated 21-month-old CA/CPR group, EC-treated animals displayed up-regulated NRG1 and ErbB4 expression, lowered NF-κB phosphorylation and elevated BCL2 levels. As the above findings demonstrate, long-term oral administration of EC reinforces resistance to CIRI in aged rats, potentially by promoting NRG1-ErbB4 interaction and attenuating NF-κB-mediated inflammatory and apoptotic responses. As a member of the EGF ligand family, NRG1 not only facilitates connections between cells, but also conducts a role in the maturation and development of tissue cells. It predominantly interacts with ErbB receptors to NRG1-ErbB dimmers, thereby activating downstream signaling cascades that orchestrate cellular growth and proliferative processes. As relevant study has substantiated, the anti-inflammatory properties of NRG1 stem from a reduction in proinflammatory gene expression in microglia and differential modulation of the NF-κB signaling pathway [39]. These findings corroborate prior research documenting cognitive enhancement following NRG1 administration and age-related downregulation of NRG1 expression in aged rodents [40]. Accumulating evidence supports a protective role of NRG1 signaling in neuroinflammation, particularly in Alzheimer’s disease (AD) models [41]. The anti-inflammatory properties of NRG1 are bound up with its capacity to suppress proinflammatory gene expression induced by ischemia through NF-κB pathway modification [42]. NF-κB, a central regulator of inflammation, exerts a significant influence on the pathophysiology of ischemic stroke. The phosphorylation and degradation of IκBα in the classical NF-κB pathway give rise to the nuclear translocation of the p65/p50 heterodimer, which stimulates the generation of proinflammatory cytokines such as TNF-α, IL-1β and IL-6 [43]. Cerebral ischemia triggers NF-κB activation, thereby initiating an inflammatory cascade in the central nervous system. Brain injury is exacerbated by proinflammatory cytokines, which in turn bring about the release of more inflammatory and neurotoxic mediators from immune and glial cells. As evidently suggested by our findings, EC therapy lowered IκBα and p65 phosphorylation levels, demonstrating that EC may inhibit NF-κB activation by reinforcing the NRG1/ErbB4 signaling pathway. As a consequence, changes in the observed results may be spawned from variations in the experimental models. Aside from that, this research has demonstrated that NRG1 controls microglial polarization through the NF-κB pathway, which not only lessens neuroinflammation, but also strengthens cognitive performance [39]. Crucially, EC’s modulation of NRG1 diverges from canonical anti-aging compounds (e.g., resveratrol’s SIRT1 activation), suggesting a novel pathway for geroprotection [44]. Furthermore, in vitro and in vivo models of ischemia injury have suggested neuroprotective effects as a consequence of NRG1 intracellular signaling [45]. A prior study confirmed that NRG1 lowers DNA fragmentation, caspase-3 immunoreactivity and cortical infarct size subsequent to middle cerebral artery occlusion (MCAO), which sufficiently underlines its enormous potential to ameliorate neurological recovery by inhibiting apoptosis [46]. The NRG1/ErbB4 signaling pathway is involved in diverse array of fundamental functions, comprising cognition, neuronal migration [47], and anti-inflammatory responses [48].

Of course, there are limitations to our study. Several limitations merit consideration. First, single-timepoint assessment at 24 h post-CPR may overlook dynamic changes in inflammatory cascades; longitudinal studies up to 7 d are needed. Second, while EC improved neurological scores, cognitive–behavioral outcomes were unexamined—a critical gap given CA/CPR survivors’ high cognitive impairment rates. Third, the cell-type-specific role of NRG1 requires conditional knockout models. Future work should explore EC’s synergy with therapeutic hypothermia, the current clinical gold standard for post-cardiac arrest (CA) neuroprotection, while also integrating long-term behavioral assessments (such as the water maze test 7 days after CA) and investigating the relationship between microglia and aging-related inflammation.

## 5. Conclusions

Collectively, our investigation established a physiologically aging rat model with subsequent induction of CA/CPR. Our findings suggest that EC inhibited inflammatory aging and alleviated post-CA/CPR brain injury in aged rats potentially through the suppression of the NRG1-NF-κB signaling pathway, and has enormous potential to position it as a promising oral anti-aging health care drug that is both food and medicine.

Although these findings offer preliminary evidence connecting EC’s anti-inflammatory geroprotective effects to NRG1-NF-κB pathway modulation, comprehensive mechanistic elucidation necessitates further pharmacological investigation. The time point of 24 h post-CPR was selected, grounded in the peak severity of brain injury; long-term effects following cerebral ischemia–reperfusion beyond 24 h remain imperative for comprehensive therapeutic exploration.

## Figures and Tables

**Table 1:** Rat CPR duration and survival rate in 24 h after resuscitation. A ratio was employed to denote the survival rate, while the mean ± standard deviation was utilized to represent the CPR time. * *p* < 0.05 vs. 3 Mo_CA/CPR_Veh group.

Group	Survival Rat	*n* (Final Survival Quantity)	CPR Duration
3 Mo_Sham_Veh	6/6 (100%)	6	-
21 Mo_Sham_Veh	6/6 (100%)	6	-
21 Mo_Sham_EC1	9/9 (100%)	9	-
21 Mo_Sham_EC2	10/10 (100%)	10	-
3 Mo_CA/CPR_Veh	6/8 (75%)	6	99.83 ± 14.69
21 Mo_CA/CPR_Veh	6/14 (42.9%) *	6	96.00 ± 14.79
21 Mo_CA/CPR_EC1	6/11 (54.5%)	6	106.5 ± 19.90
21 Mo_CA/CPR_EC2	6/10 (60%)	6	106.83 ± 14.29

## Data Availability

The original contributions presented in this study are included in the manuscript/Supplementary Materials. Further inquiries can be directed at the corresponding author.

## Supplementary Materials

The following supporting information can be downloaded at: https://www.mdpi.com/article/10.3390/cimb47100793/s1.
