# Supplementary material for: Epicatechin Protects Against Post-Cardiac Arrest Brain Injury in Aged Rats via NRG1-Mediated Suppression of Neuroinflammation
Source: Curr Issues Mol Biol. 2025 Sep 24;47(10):793. doi: 10.3390/cimb47100793 (PMC12562848; doi:10.3390/cimb47100793)
Supplement: Supplementary file 1 [file cimb-47-00793-s001.zip › cimb-3865264-supplementary.pdf]

Supplementary Tables

Table S1. The table lists the experimental groups and their short names.

Number	Experimental Groups (Number of Animals)	Short Names of Experimental Groups
1	3-Month-old young Sham group($n = 6$)	3 Mo_Sham_Veh
2	21-Month-old water Sham group($n = 6$)	21 Mo_Sham_Veh
3	21-Month-old EC 1 mg/kg Sham group($n = 9$)	21 Mo_Sham_EC1
4	21-Month-old EC 2 mg/kg Sham group($n = 10$)	21 Mo_Sham_EC2
5	3-Month-old young CA/CPR group($n = 8$)	3 Mo_CA/CPR_Veh
6	21-Month-old water CA/CPR group($n = 14$)	21 Mo_CA/CPR_Veh
7	21-Month-old EC 1 mg/kg CA/CPR group($n = 11$)	21 Mo_CA/CPR_EC1
8	21-Month-old EC 2 mg/kg CA/CPR group($n = 10$)	21 Mo_CA/CPR_EC2

Table S2. qPCR primers for target genes.

Number	Name	Sense(5′to3′)	Antisense(5′to3′)
1	NRG1	CCAGCAACTCAACTCCTTTCATC	GGTCGTCTCGTACTCCTCATCC
2	ERbB4	AAAGGCGGCTTGTTCATCG	TGCCACCGTCAGCATTGTAT
3	IKK-α	TGCAGAGTCAGGACCGAGTTC	TACTGAGGGCCACTTCCACC
4	NF-KBp65	CGTGAGGCTGTTTGGTTTGA	GTCTTATGGCTGAGGTCTGGTCT
5	p21	CCTGTCTCCGCTCAGATTGTAA	ACCAGCTTTGGGATAGGGTGTA
6	p53	AATCCAAGGAAAGCCTGAAGC	AAGCGGCAGAAATGTAAATGTG
7	β-actin	GGAGATTACTGCCCTGGCTCCTA	GACTCATCGTACTCCTGCTTGCTG

Supplementary Figure

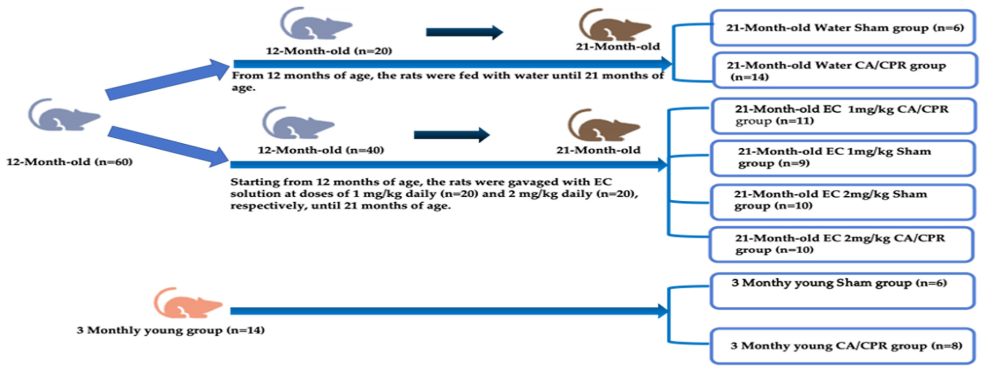

Figure S1. The grouping of 21-month-old and 3-month-old SD rats for CA/CPR and the number in each group. Pink: 3-Month-old young group; Gray: 12-Month-old group; Brown: 21-Month-old; Blue arrow: Random grouping.
